# Downregulation of *SMIM3* inhibits growth of leukemia via PI3K-AKT signaling pathway and correlates with prognosis of adult acute myeloid leukemia with normal karyotype

**DOI:** 10.1186/s12967-022-03831-8

**Published:** 2022-12-22

**Authors:** Yu Liu, Yufei Chen, Yajun Liu, Mengya Li, Yu Zhang, Luyao Shi, Lu Yang, Tao Li, Yafei Li, Zhongxing Jiang, Yanfang Liu, Chong Wang, Shujuan Wang

**Affiliations:** 1 Department of Hematology, The First Affiliated Hospital of Zhengzhou University, No. 1 Jianshe East Road, Erqi District, Zhengzhou, 450052 China; 2 Department of Orthopaedics, Warren Alpert Medical School/Rhode Island Hospital, Brown University, Providence, Rhode Island USA

**Keywords:** Acute myeloid leukemia, Prognosis, *SMIM3*, Cell proliferation, Cell cycle, PI3K-AKT

## Abstract

**Background:**

Acute myeloid leukemia (AML) patients with normal karyotype (NK-AML) have significant variabilities in outcomes. The European Leukemia Net stratification system and some prognostic models have been used to evaluate risk stratification. However, these common standards still have some limitations. The biological functions and mechanisms of Small Integral Membrane Protein 3 (*SMIM3*) have seldomly been investigated. To this date, the prognostic value of *SMIM3* in AML has not been reported. This study aimed to explore the clinical significance, biological effects and molecular mechanisms of *SMIM3* in AML.

**Methods:**

RT-qPCR was applied to detect the expression level of *SMIM3* in bone marrow specimens from 236 newly diagnosed adult AML patients and 23 healthy volunteers. AML cell lines, Kasumi-1 and THP-1, were used for lentiviral transfection. CCK8 and colony formation assays were used to detect cell proliferation. Cell cycle and apoptosis were analyzed by flow cytometry. Western blot was performed to explore relevant signaling pathways. The biological functions of *SMIM3* in vivo were validated by xenograft tumor mouse model. Survival rate was evaluated by *Log-Rank* test and *Kaplan–Meier. Cox* regression model was used to analyze multivariate analysis. The correlations between *SMIM3* and drug resistance were also explored.

**Results:**

Through multiple datasets and our clinical group, *SMIM3* was shown to be significantly upregulated in adult AML compared to healthy subjects. *SMIM3* overexpression conferred a worse prognosis and was identified as an independent prognostic factor in 95 adult NK-AML patients. Knockdown of *SMIM3* inhibited cell proliferation and cell cycle progression, and induced cell apoptosis in AML cells. The reduced *SMIM3* expression significantly suppressed tumor growth in the xenograft mouse model. Western blot analysis showed downregulation of p-PI3K and p-AKT in *SMIM3*-knockdown AML cell lines. *SMIM3* may also be associated with some PI3K-AKT and first-line targeted drugs.

**Conclusions:**

*SMIM3* was highly expressed in adult AML, and such high-level expression of *SMIM3* was associated with a poor prognosis in adult AML. Knockdown of *SMIM3* inhibited the proliferation of AML through regulation of the PI3K-AKT signaling pathway. *SMIM3* may serve as a potential prognostic marker and a therapeutic target for AML in the future.

**Supplementary Information:**

The online version contains supplementary material available at 10.1186/s12967-022-03831-8.

## Background

Acute myeloid leukemia (AML) is a genetically and clinically heterogeneous hematologic malignancy [1] with a 3.7/100 000 incidence per year [2]. Nearly 80% of adult acute leukemia are AML [3], and the 5-year overall survival rate for AML patients older than 60 years is about 25% [4]. The application of various first-line chemotherapeutic agents, molecular-targeted agents, immunotherapeutic agents and hematopoietic stem cell transplantation (HSCT) has dramatically improved the treatment outcomes of AML patients [5, 6]. However, the relapse rate of AML patients is 80% [7], and the mortality rate has been over 50% [8]. Stratified diagnosis and treatment of AML have become the focus of research in recent years. The classification of AML is based on cytogenetic and mutational profiles. Moreover, some additional pre-disposing features are considered as prognostic factors, which include therapy-related, prior myelodysplastic syndrome (MDS) or MDS/myeloproliferative neoplasm [MPN], and germline predisposition [9]. Also, the response to initial therapy and assessment of early minimal residual disease (MRD) is also crucial in risk classification[9]. Based on the existing risk-stratification system, patients in different risk strata accept the corresponding treatment. Even so, there is still a chance of drug resistance and relapse in low- and intermediate-risk patients.

Karyotyping is crucial in the risk stratification of AML. It was reported that 40–50% AML patients have a normal karyotype (NK) [10]. Single genes, such as *CEBPA* [11], *FLT3-ITD* [12, 13] and *NPM1 *[13], could provide references for predicting the prognosis of NK-AML patients. It used to be considered that patients with NK-AML had a medium prognosis. However, it was found that NK-AML patients with high-risk mutated genes or aberrantly expressed genes had poor prognosis [14, 15]. This situation suggested that the risk stratification of NK-AML patients required further improvements. Gene expression-based scoring systems have potentials in predicting the prognostic value in AML [16]. Therefore, novel prognostic biomarkers are highly anticipated to improve the risk stratification for NK-AML.

Based on the ImmuCo database and clinical specimens, we screened seven genes associated with acute leukemia as candidate prognosis biomarkers. In the follow-up verification, we found that the expression level of Small Integral Membrane Protein 3 (*SMIM3*, also called Nid67*)* in AML was significantly higher than that in normal controls. SMIM families contain multiple members. Small integral membrane protein 1 (*SMIM1*), a tail-anchored transmembrane protein [17], is associated with Vel-negative blood type [18, 19]. *SMIM1* also could influence red blood cell traits [20]. A study revealed that *SMIM4* is a respiratory chain assembly factor [21]. *SMIM20* is expressed in the adult brain, and may function in fertility and reproduction [22]. A study suggested that *SMIM20* could be a new target of endometriosis [23]. Sha Liu et al. found that *Lnc-SMIM20-*1 upregulation is associated with poor prognosis in AML [24]. In addition, *SMIM30* could promote the progression of hepatocellular carcinoma [25, 26].

The *SMIM3* gene is located on chromosome 5q33.1, coding a single-pass transmembrane protein consisting of 60 amino acids with a total molecular weight of 6593 Da. It is expressed in various tissues, with the highest expression in heart, ovarian and adrenal glands, and the lowest expression in skeletal muscle and cerebellum [27]. It may play a role in cell channel regulation and be associated with neuronal differentiation [27]. There are few studies on *SMIM3*, and certainly not have been studied in AML. Thus, large gaps remain to be filled in our understanding of the function and mechanism of *SMIM3* in AML*.* Currently, *SMIM3*-related hematological disease is 5q- syndrome of MDS. A study showed that a gene or genes in the Cd74 to Nid67 interval might be associated with MDS [28]. However, the mechanism has not been studied in details. In addition, *SMIM3* can be used as a sensitive and specific biomarker of radiation exposure in the radiation emergency department, and patients with expression of *SMIM3* had a poor prognosis [29]. Weining Wang et al. found that eleven genes including *SMIM3* in NCCS (n = 36) and TCGA (n = 40) databases were associated with poor overall survival rate in oral squamous cell carcinoma patients without nodal metastases [30]. However, more evidence is needed to prove whether *SMIM3* is a prognostic biomarker in oral squamous cell carcinoma.

In this study, we examined the expression and prognostic value of *SMIM3* in AML. We further demonstrated the effect of *SMIM3* on cellular and biological behavior both in vitro and in vivo. Meanwhile, we investigated the mechanism of *SMIM3* regulation. We also correlated the *SMIM3* expression with targeted therapy responsiveness.

## Methods

### Database analyses

We collected and compared the *SMIM3* expression data of 264 hematopoietic stem cell (HSC) samples and 814 AML bone marrow mononuclear cells (BMMC) in the ImmuCo database[31] (http://immuco.bjmu.edu.cn). Also, 8295 normal samples and 9807 tumor samples in UCSC XENA (https://xena.ucsc.edu), Genotype-Tissue Expression (GTEx) (http://commonfund.nih.gov/GTEx/) and The Cancer Genome Atlas (TCGA) database (https://tcga-data.nci.nih.gov/tcga/) were compared (including 173 AML samples). In the Gene Expression Omnibus (GEO) database (http://www.ncbi.nlm.nih.gov/geo/), GSE12417 -GPL97 and GSE12417-GPL570 [32] were used for gene expression and survival analyses.

### Subjects

The 236 bone marrow samples from newly diagnosed AML patients and 23 samples from healthy donors were enrolled at the First Affiliated Hospital of Zhengzhou University between February 2017 and March 2022. The exclusion criteria were patients who didn’t treat or treat elsewhere and patients with acute promyelocytic leukemia. The inclusion criteria was patients who accepted at least one course of treatment. We referred to this cohort as the ZZU cohort. Clinical information acquired from patient medical records mainly included gender, age, white blood cell count (WBC), hemoglobin (HGB), platelet (PLT), peripheral blasts (PB), bone marrow (BM) blasts at diagnosis, fusion gene, gene mutations and chromosomal karyotype, risk stratification, treatment regimens, transplant, and survival status. The induction therapy contained IA and DA regimens: standard-dose cytarabine (Ara-C) 100–200 mg·m^− 2^·d^− 1^ × 7 d combined with idarubicin 10–12 mg·m^− 2^·d^− 1^ × 3d or daunorubicin (DNR) 60 mg·m^− 2^·d^− 1^ × 3d. After remission, patients accepted high-dose Ara-C 3 g/m^2^, every 12 h × 3d. Patients without HSCT accepted four courses. Patients with HSCT accepted two courses, and then they accept HSCT. Patients over 60 years old or patients who cannot tolerate intensive chemotherapy accepted chemotherapy with demethylating drugs ± CAG ± venetoclax regimen until progression. Subjects were followed up until death, loss to follow-up or March 2022. The diagnosis of AML, complete remission (CR), relapse, risk stratification and overall survival (OS) were defined according to NCCN guideline for acute myeloid leukemia Version 2.2021[33]. The study was approved by the Ethics Committee of the First Affiliated Hospital of Zhengzhou University and informed consent was obtained according to the Declaration of Helsinki.

### Next-generation sequencing

Next-Generation sequencing was applied to assess the mutational hotspots of genes. Based on an Illumina MiSeq System (Illumina, San Diego, CA) high-throughput sequencing platform, a Rightongene AML/MDS/MPN Sequencing Panel (Rightongene, Shanghai, China) was applied to finish the detection. Details of the variant calling, filtering, and annotation are shown in the published reports [34].

### Cytogenetics and fusion genes analysis

Based on the International System for Human Cytogenetic Nomenclature, chromosomal banding analyses were performed by G-banding techniques. Real-time quantitative polymerase chain reaction (RT-qPCR) was performed to detect fusion genes with Multiplex RT-qPCR Fusion Gene Kits (Rightongene, Shanghai, China).

### Cell lines and reagents

The human AML cell line Kasumi-1 was purchased from American Type Culture Collection (Manassas, VA, USA). The human AML cell line THP-1 was purchased from the cell bank of the Chinese Academy of Sciences (Shanghai, China). The cell lines were cultured in 90% Roswell Park Memorial Institute (RPMI**)** 1640 supplemented with 10% Fetal bovine serum (FBS) and 1% penicillin/streptomycin (P/S) (all from Gibco, Billings, MT, USA). The culture conditions were 37 °C, 5% CO_2_, and 95% humidity. SC79 (Beyotime, Shanghai, China) was used as an AKT activator.

### Lentiviral transduction

Lentiviral shRNA transduction was performed in Kasumi-1 and THP-1 cells with human *SMIM3* shRNA lentiviral particles (Genechem, Shanghai, China) or empty control lentiviral particles (Genechem, Shanghai, China). All infections were done at a multiplicity of infection (MOI) of 100. At 12 h post-transfection, media containing lentiviral particles was replaced with fresh complete medium. Stably transfected Kasumi-1 and THP-1 cells were selected with 2 mg/ml puromycin dihydrochloride (Genechem, Shanghai, China) at 72 h post-infection. Stable *SMIM3*-knockdown cells and control cells were acquired 4 weeks after antibiotic selection. The expression level of *SMIM3* was confirmed by RT-qPCR and the immune fluorescence (IF) technique.

### RNA extraction and RT-qPCR

Bone marrow samples were collected into Ethylene Diamine Tetraacetic Acid (EDTA) anticoagulant–containing tubes. Mononuclear cells were obtained via density gradient centrifugation. Total RNA was extracted using TRIzol Reagent (Invitrogen, Carlsbad, CA, USA). The cDNA was synthesized using a High Capacity cDNA Reverse Transcription Kit (Applied Biosystems, Foster City, CA, USA) [35]. *SMIM3* transcript levels were detected by the Taqman method using RT-qPCR as previously described [35]. Serial dilutions of plasmids expressing *SMIM3* and *ABL1* (Genechem, Shanghai, China) were amplified to construct standard quantification curves. The *SMIM3* and *ABL1* copy numbers were calculated from standard curves using Ct values. The *SMIM3* transcript level was calculated as the ratio of the *SMIM3* copy number/*ABL1* copy number as previously described [36].The primers and probe sequences of *SMIM3* and *ABL1* are shown in Additional file 1: Table S1.

### Immune fluorescence analysis

The cells in T25 flasks were collected and washed several times with phosphate-buffered saline (PBS). Then the cells were fixed using 4% paraformaldehyde (PFA) for 20 min at room temperature (RT). Further, Triton-X-100 (Beyotime, Shanghai, China) was applied to permeabilized cells for 10 min, and nonspecific binding was blocked with 5% BSA (Solaibao Biotechnology, Beijing, China) for 30 min at RT. Followed by washing, cells were incubated overnight at 4 °C with diluted (1:100) primary anti-*SMIM3* Polyclonal Antibody (Thermo Fisher Scientific, Waltham, MA, USA). The cells were then incubated with diluted (1:200) secondary antibody Cy3 conjugated Goat Anti-Rabbit IgG (H + L) (Servicebio, Wuhan, China) for 1 h at RT, followed by washing in PBS and staining with DAPI (Solaibao Biotechnology, Beijing, China). Analysis was conducted under a confocal laser scan microscope (Zeiss, Oberkochen, Germany).

### Western blot analyses

RIPA lysis buffer (Beyotime, Shanghai, China) supplemented with Protein phosphatase inhibitor (Biomed, Beijing, China) and phenylmethylsulfonylfluoride (PMSF, Biomed) was used for protein extraction. Lysates were run on 10% polyacrylamide gel electrophoresis (PAGE) gels, and protein bands were transferred to 0.45 μm polyvinylidene difluoride (PVDF) membrane (Millipore, Billerica, MA, USA), then blocked with 5% skim milk at room temperature for 2 h. The membrane was incubated with primary antibodies (GAPDH, cleaved-PARP, cleaved caspase3, p27 Kip1, Cyclin D1, CDK4, p-AKT, AKT, PI3K, Cell Signaling Technology [CST], MA, USA, 1:1000; p-PI3K, Affinity Biosciences LTD, Jiangsu, China, 1:1000) overnight at 4 °C and probed with secondary antibodies (goat anti-rabbit IgG horseradish peroxidase (HRP), Zhongshan Golden Bridge Biotechnology, Beijing, China, 1:2000) at room temperature for 1 h. The immunoreactive bands were detected using Super ECL Prime (US EVERBRIGHT, Suzhou, China) according to the manufacturer’s protocol.

### Cell proliferation

Cell proliferation was measured through Cell Counting Kit-8 (CCK8, Dojin Laboratories, Kumamoto, Japan) assay. CCK8 assay was carried out according to the standard protocol by seeding cells in a 96-well plate at a density of 1 × 10^5^ cells/well. Then 10 μl of the kit reagent was added into each well after 0, 24, 48, 72, 96 h. After incubation for 3 h, the absorbance was measured at 450 nm spectrophotometrically. The experiments were performed in triplicate.

### Colony forming assay

To analyze the colony formation, we seeded cells in 35 mm dishes at 6 × 10^3^ cells per well in methylcellulose-based MethoCult medium (STEMCELLTM TECHNOLOGIES, Vancouver, British Columbia, Canada). The surviving colonies (≥ 30 cells per colony) were counted under an inverted microscope after 10 days of growing in a humid incubator. All experiments were performed 3 times independently.

### Cell cycle and apoptosis analyses

The cells were inoculated in six-well plates with a density of 1 × 10^5^ cells per ml. Then they were synchronized via serum starvation (grown in RPMI 1640 without FBS). After 24 h, the medium was replaced with complete medium for an additional 72 h. The Cell Cycle Staining Kit (Lianke Biotechnology, Hangzhou, China.) was used for cell cycle analyses and the Annexin V-APC/PI Apoptosis Kit (US EVERBRIGHT) was applied for the apoptosis assay. The cell cycle and apoptosis were examined by BD FACSCelesta^™^ flow cytometry (*BD* Biosciences, California, USA).

### Xenograft tumor mouse model

Xenograft model experiments were conducted using 6-week-old male BALB/c nude mice (Beijing HFK Bioscience Co., Ltd.; Beijing, China). All mice were divided into three groups (CTRL, KD1 and KD2), each group consisted of 3 mice. After 2 days of cyclophosphamide intraperitoneal injection (100 mg/kg/d × 2d), the transfected cells were syringed in the mice’s right flank. Tumor volume was calculated every other day for 14 or 18 days using the following formula: volume (mm^3^) = (L*I^2^)/2, where L and I are the lengthiest and shortest diameters, respectively. Subsequently, all mice were euthanized and the xenograft tumors were harvested, weighed, and photographed. This study was approved by the Ethics Committee of the First Affiliated Hospital of Zhengzhou University.

### H&E staining

The tissue was fixed in 10% formaldehyde. Firstly, the paraffin sections were deparaffinized. Then the sections were stained with Hematoxylin and Eosin staining solutions. After that, the sections were dehydrated with gradient ethanol, transparentized using xylene. Finally, neutral resin was applied to seal the sections.

### Immunohistochemical assays

Tissue was also fixed in 10% formaldehyde. According to the standard protocol, the paraffin sections were deparaffinized. After repairing antigen and blocking endogenous peroxidase act, tissue sections were blocked in 3% BSA. Tissue sections were incubated with primary antibodies (Ki67, cleaved caspase3, cleaved PARP1, Servicebio, Wuhan, China) at 4 °C overnight, followed by conjugated secondary antibodies (Servicebio, Wuhan, China) and diaminobenzidine (DAB, Servicebio, Wuhan, China). Then nuclei counterstaining was performed with Mayers hematoxylin (Servicebio, Wuhan, China). Finally, the tissue sections were dehydrated and sealed.

### Statistical analyses

*Pearson Chi-square test* or Fisher exact analysis was applied for categorical data. *Student’s t-test* or *Mann–Whitney U-test* was applied for continuous variables. Survival was estimated using the *Kaplan–Meier* method and *Log-Rank* test. In the TCGA-LAML, GEO 12,417 and ZZU cohort, patients were classified into the high expression group and the low expression group according to cutoff value of *SMIM3*. A *Cox* proportional hazard regression model was used to determine associations between *SMIM3* transcript levels and OS. Variables with *P* < 0.2 in the single variable analysis were included in the model. *P* < *0.05* (two-sided) was considered significant (*, *P* < *0.05*; **, *P* < 0.01; ***, *P* < 0.001). The receiver operating characteristic (ROC) curve was generated using the R package “timeROC” [37] and “survival” (https://CRAN.R-project.org/package=survival) to evaluate the diagnostic value. The hazard ratio (*HR*) and corresponding 95% confidence interval (*CI*) were also calculated. Data analysis was performed with Graphpad Prism^™^ 8.01 (San Diego, California, USA) and R (version 4.1.1, Auckland, NZ, United States, http://www.r-project.org/).

## Results

### The SMIM3 was significantly upregulated in subjects with acute Myeloid Leukemia

**Fig. 1:**
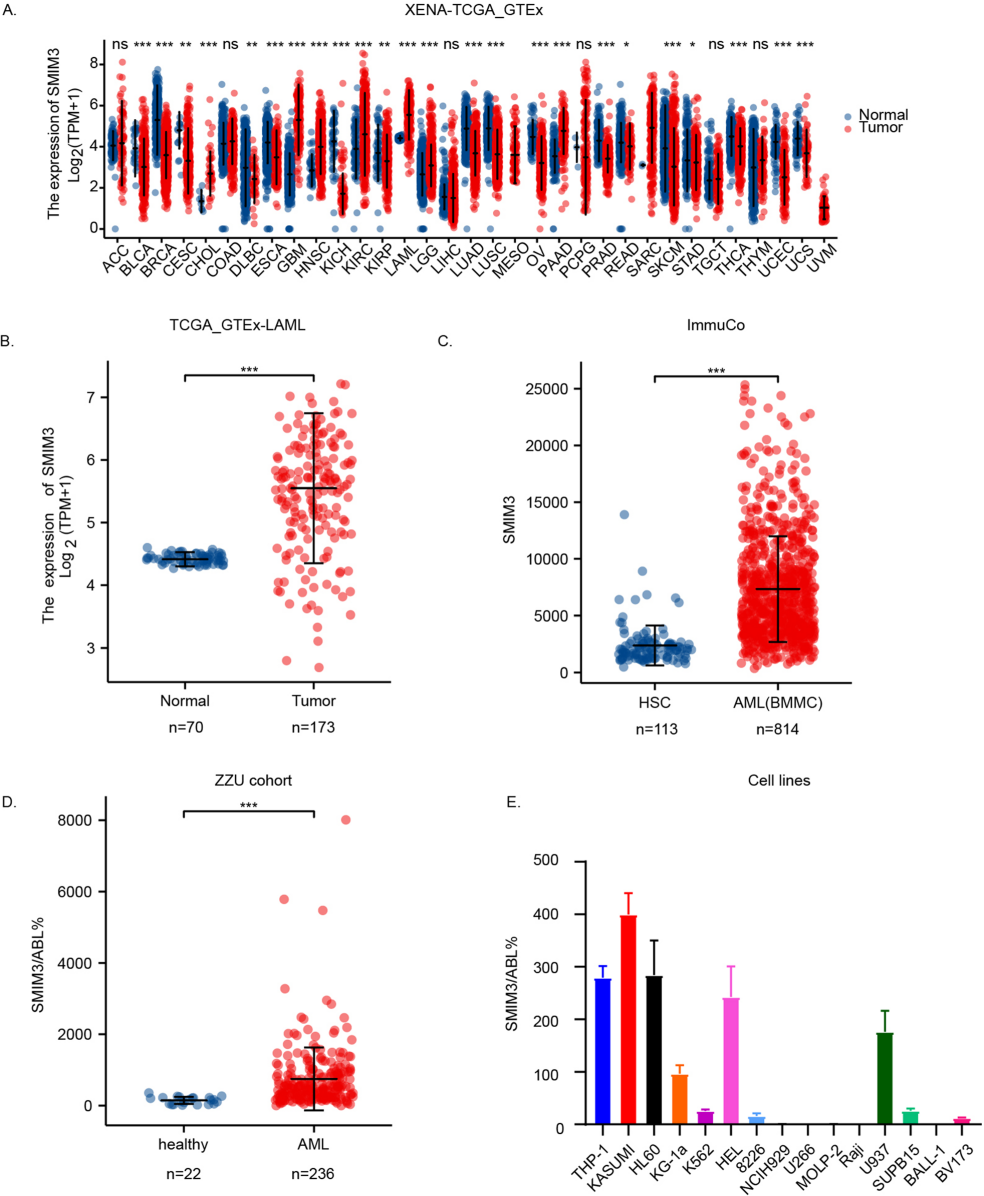
AML samples showed a higher expression of *SMIM3* compared to normal samples. **A** Expression levels of *SMIM3* in paired samples of normal and tumor patients in different cancers. **B** In the TCGA-GTEx database, *SMIM3* showed significantly higher expression in AML patients (n = 173) than in normal samples (n = 70). **C** In the ImmuCo database, the gene expression level of *SMIM3* was higher in AML bone marrow mononuclear cells (BMMC, n = 814) than in hematopoietic stem cells (HSC, n = 113). **D** AML samples (n = 236) from the ZZU cohort showed a significant increase in *SMIM3* expression compared to normal bone marrow samples (n = 22). **E** *SMIM3* expression in different cell lines. *, *P* < 0.05; **, *P* < 0.01; ***, *P* < 0.001

First, to identify the role of *SMIM3*, we examined the gene expression in multiple public databases and our cohort (ZZU cohort). We compared the expression of *SMIM3* in GTEX database, UCSC XENA and TCGA database, and results showed that *SMIM3* is significantly overexpressed in 8 cancers, including LAML, and reduced in 16 cancers (Fig. 1A, B). The ImmuCo database analysis showed that the expression level of *SMIM3* was higher in AML(BMMC) than in hematopoietic stem cell (*P* < 0.0001, Fig. 1C). Then we investigated the mRNA transcript levels of *SMIM3* in the bone marrow of newly diagnosed subjects with AML and normal healthy individuals in the ZZU cohort. The expression of *SMIM3* in 236 AML patients was significantly higher than in 22 healthy individuals (median 501.55%, Inter Quartile Range *IQR* [264.632–913.75%] vs 160.775%, *IQR* [50.741–220.642%], *P* < 0.001, Fig. 1D).

### The expression of SMIM3 was associated with AML prognosis

**Fig. 2:**
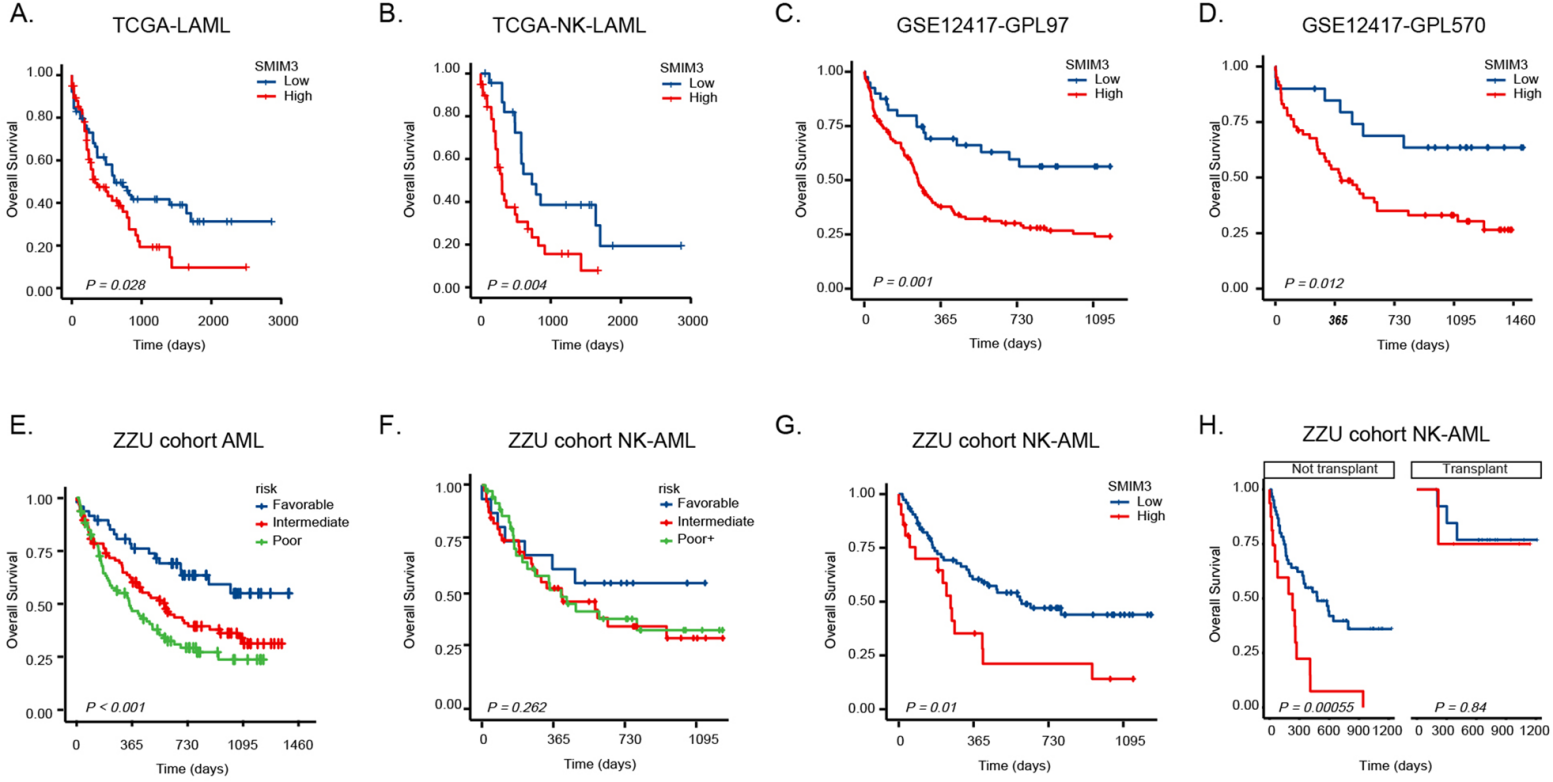
Overall survival (OS) of adult subjects with AML according to *SMIM3*. **A** OS of 151 AML subjects in TCGA. **B** OS of 69 AML subjects with normal karyotype in TCGA. **C** OS of 163 AML subjects in GSE12417-GPL97. **D** OS of 79 AML subjects in GSE12417-GPL570. **G** OS of 95 AML subjects with normal karyotype in ZZU cohort. **H** Differences in OS between the transplant and non-transplant groups in ZZU NK-AML cohort. Overall survival (OS) of adult subjects with AML according to risk-stratification system. **E** OS of 236 AML subjects in ZZU cohort. **F** OS of 95 AML subjects with normal karyotype in ZZU cohort

Subsequently, we analyzed the role of *SMIM3* in the prognosis of AML patients. In TCGA database, patients with high *SMIM3* expression showed a worse 2-year OS than those with low *SMIM3* expression (50.1% [39.4%-63.6%] vs. 36.1% [25.1–51.9%]; *P* = 0.018; Fig. 2A). Moreover, in 69 AML with normal karyotype (NK-AML), patients with high *SMIM3* expression had a worse 2-year OS than those with low *SMIM3* expression (41.1% [26.3–64.0%] vs. 32.8% [18.8–57.0%]; *P* = 0.046; Fig. 2B). The prognostic value of *SMIM3* in NK-AML was also validated in the GSE12417 cohort (GPL97 and GPL570). All AML samples in this cohort had normal karyotype. As previously found in the TCGA cohort, H-*SMIM3* groups were associated with poor survival outcome (Fig. 2C, D).

In ZZU AML cohort, the present risk stratification had prognostic significance in the entire cohort (Fig. 2E), but not in the normal karyotype-AML (NK-AML) group (Fig. 2F). In the whole cohort, the 2-year OS between the low expression group and the high expression group showed no significant difference (42.2%, 95%*CI* [35.5–50.2%] vs 35.8% [22.2–57.9%], *P* = 0.186; Additional file 2: Figure S1). However, in the NK-AML subgroup (n = 95), subjects with low *SMIM3* expression showed a favorable 2-years OS compared with subjects with high *SMIM3* expression (47.0%, 95%*CI* [36.3–60.9%] vs 21.1% [8.1–54.9%], P = 0.01; Fig. 2G). The main clinical characteristics of NK-AML in the ZZU cohort were shown in Additional file 1: Table S2. Multivariate *Cox* regression showed that *SMIM3* and transplant were independently associated with OS (Table 1). We then performed a subgroup analysis in patients with or without transplant, and the result showed that low *SMIM3* group had a favorable OS (Fig. 2H, P < 0.001) in patients without transplant, but showed no prognostic impact in patients with transplant (Fig. 2H, P = 0.84) (Additional file 3).

**Table1:** Univariate and Multivariate Analysis of Overall Survival in AML with Normal Karyotype

characteristics	Univariate analysis		Multivariable analysis	
	*HR* (95%*CI*)	*P*-value	*HR* (95%*CI*)	*P-*value
**SMIM3**	**2.19(1.19–4.01)**	**0.011**	**2.9 (1.51–5.57)**	**0.001**
Age	2.07(1.11–3.85)	0.022	1.58 (0.82–3.02)	0.171
Sex	0.65(0.37–1.14)	0.132	0.74 (0.41–1.32)	0.308
WBC, × 10^9^/L	0.81(0.47–1.41)	0.464	NA	NA
PLT, × 10^9^/L	1.91(1.09–3.33)	0.023	1.56(0.81–2.98)	0.18
LDH U/L	1.39(0.78–2.49)	0.264	NA	NA
PB(%)	0.56 (0.28–1.09)	0.087	0.94(0.43–2.08)	0.884
*FLT3*	1.09(0.6–1.96)	0.784	NA	NA
*FLT3-ITD*	1.13(0.63–2.04)	0.678	NA	NA
*FLT3-TKD*	0(0-Inf)	0.997	NA	NA
*RUNX1*	2.39(0.73–7.79)	0.148	1.56(0.45–5.49)	0.485
*ASXL1*	1.03(0.54–1.96)	0.94	NA	NA
*CBFβ*	NA	NA	NA	NA
*CBL*	2.2(0.53–9.12)	0.278	NA	NA
*CEBPA*	0.67(0.35–1.27)	0.221	NA	NA
*DNMT3A*	0.93(0.47–1.86)	0.835	NA	NA
*ETO*	NA	NA	NA	NA
*ETV6*	0.64(0.09–4.66)	0.662	NA	NA
*EZH2*	0(0-Inf)	0.996	NA	NA
*IDH1*	0.68(0.24–1.89)	0.459	NA	NA
*IDH2*	0.47(0.12–1.95)	0.302	NA	NA
*JAK2*	0(0-Inf)	0.996	NA	NA
*KIT*	0(0-Inf)	0.996	NA	NA
*MLL*	2.65(0.36–19.52)	0.339	NA	NA
*NPM1*	1.12(0.61–2.08)	0.708	NA	NA
*NRAS*	0.68(0.29–1.58)	0.367	NA	NA
*PHF6*	1.39(0.19–10.19)	0.745	NA	NA
*SETBP1*	1.77(0.43–7.3)	0.431	NA	NA
*SF3B1*	NA	NA	NA	NA
*SRSF2*	1.16(0.28–4.8)	0.842	NA	NA
*TET2*	0.72(0.42–1.24)	0.235	NA	NA
*TP53*	0(0-Inf)	0.996	NA	NA
*U2AF1*	1.08(0.46–2.53)	0.857	NA	NA
*WT1*	0.92(0.52–1.63)	0.855	NA	NA
*ZRSR2*	NA	NA	NA	NA
Risk	1.15(0.79–1.67)	0.476	NA	NA
**Transplant**	**0.23(0.08–0.64)**	**0.005**	**0.26(0.09–0.76)**	**0.014**

*WBC* white blood cell counts, *HGB* hemoglobin, *PLT* platelet, *LDH* lactate dehydrogenase, *PB* peripheral blood

### Downregulation of SMIM3 inhibited cell proliferation and colony formation in AML cells

**Fig. 3:**
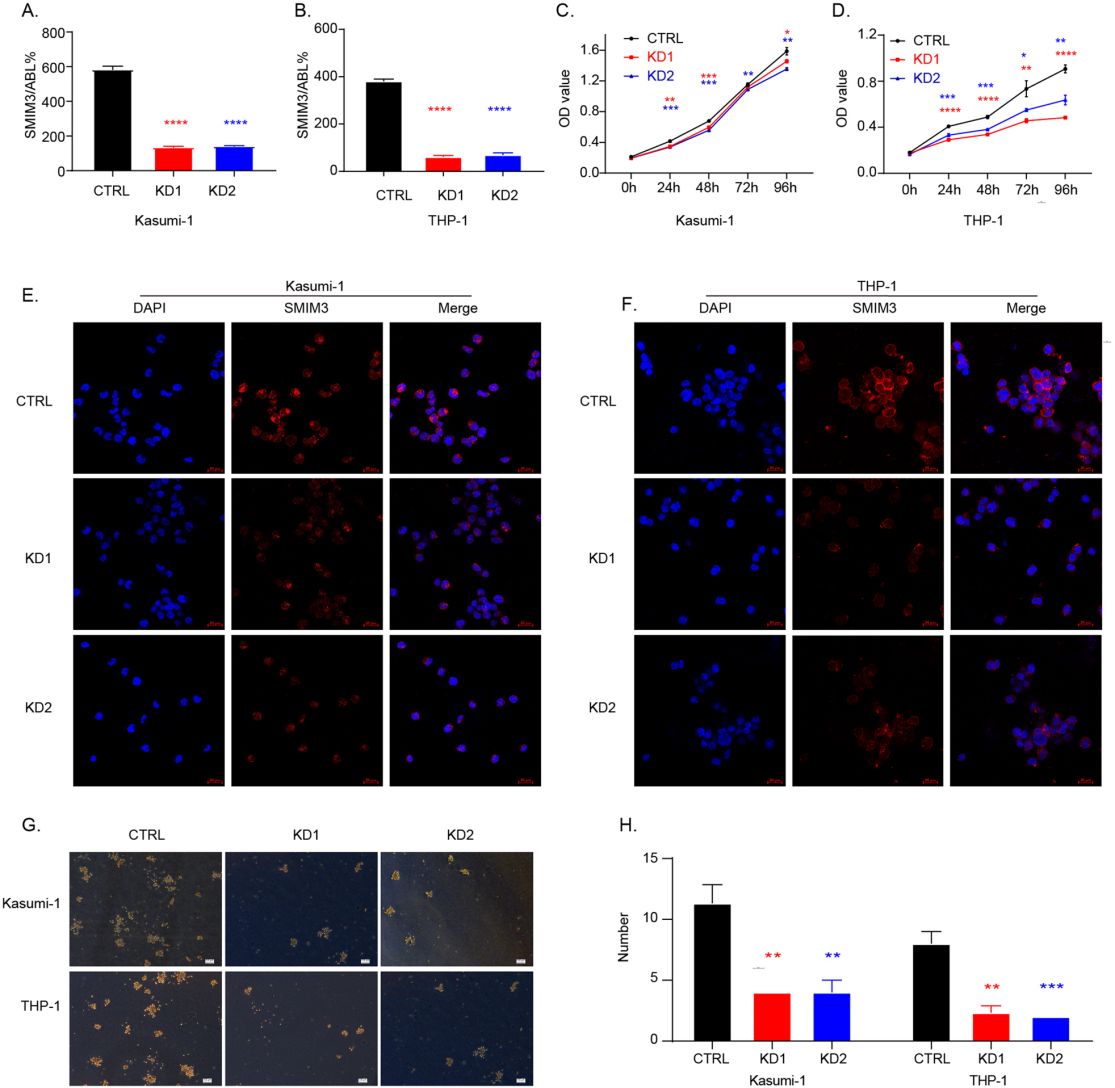
Downregulation of *SMIM3* inhibited cell proliferation and colony formation in AML cells. The efficiency of *SMIM3* knockdown in the Kasumi-1 and THP-1 cell lines was verified by **A**, **B** reverse transcription-quantitative polymerase chain reaction and **E**, **F** immune fluorescence, respectively. **C**, **D** Cell proliferation was detected by the CCK-8 assay in Kasumi-1 and THP-1. **G**, **H** The colony formation of *SMIM3*-CTRL and *SMIM3*-KD in Kasumi-1 and THP-1. CTRL, control; KD, knockdown; *P < 0.05 compared with CTRL cells; **P < 0.01 compared with CTRL cells; ***P < 0.001 compared with CTRL cells; Error bars indicate the standard deviation

To get further insight into the biological roles of *SMIM3*, in vitro experiments were performed. We then detected the transcriptional level of *SMIM3* in cell lines of hematological malignancies. RT-qPCR suggested that *SMIM3* was over-expressed in acute myeloid leukemia cell lines, while expressed with low level in lymphoma and myeloma cell lines (Fig. 1E). To further explore the role of *SMIM3* in AML, we constructed *SMIM3* knockdown stable cell lines in Kasumi-1 and THP-1 using lentiviral small hairpin RNAs. The transfection efficiency was verified by RT-qPCR (Fig. 3A, B) and Immune fluorescence (IF) (Fig. 3E, F). The results of IF demonstrated that *SMIM3* protein expression was significantly reduced compared with control group after transfection with *SMIM3* shRNA (Fig. 3E, F). Meanwhile, *SMIM3* primarily localized to the nucleus in AML cells, and was also observed in vesicles (Fig. 3E, F). CCK8 analysis showed that *SMIM3* knockdown significantly inhibited the proliferation of Kasumi-1 and THP-1 cells compared to the controls (Fig. 3C, D). Similarly, the anti-proliferative effect of *SMIM3* was observed by colony formation assays (Fig. 3G, H).

### Knockdown of SMIM3 promoted cell cycle arrest and induced apoptosis of AML cells

**Fig. 4:**
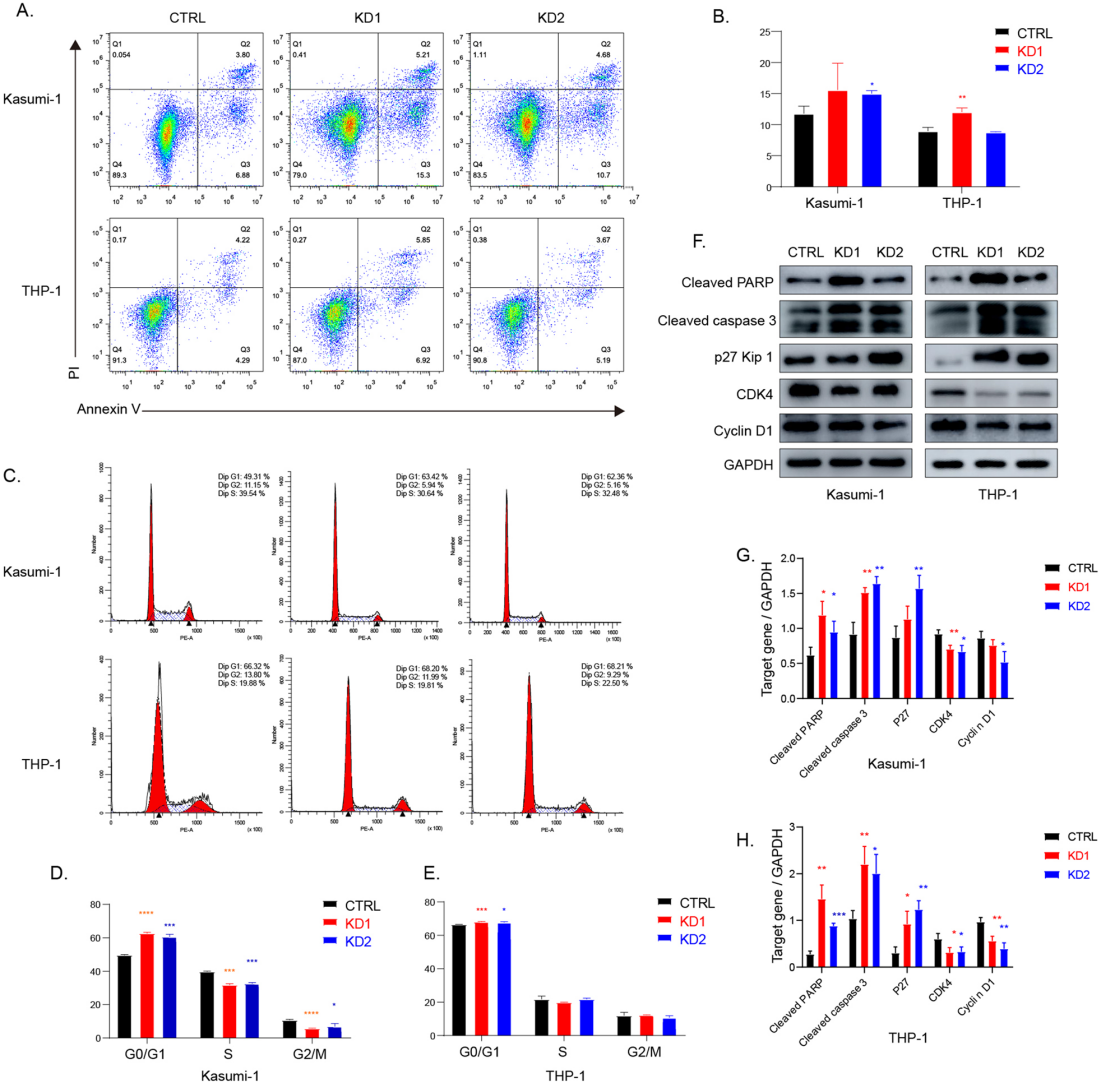
Apoptosis and cell cycle analysis in AML cell lines. **A**, **B** The flow cytometry was applied to analyze apoptosis in *SMIM3* knockdown cells compared with control. **C**–**E** Propidium iodide (PI) staining was applied to analyze the cell cycle of *SMIM3* knockdown cells compared with control. **F** Alterations in apoptosis and cell cycle-related protein assay in *SMIM3* knockdown cells compared with control. **G**, **H** Densitometric analysis of the WB signals. CTRL, control; KD, knockdown; *P < 0.05 compared with CTRL cells; **P < 0.01 compared with CTRL cells; ***P < 0.001 compared with CTRL cells

To clarify the role of *SMIM3* in cell proliferation and apoptosis, flow cytometry was performed. Results revealed that compared to the control group, knockdown of *SMIM3* significantly increased the total apoptosis rate in both Kasumi-1 and THP-1 cells (Fig. 4A, B). Moreover, knockdown of *SMIM3* increased cell counts in the G0/G1 phase in both Kasumi-1 and THP-1, and decreased cell counts in the S and G2/M phase in Kasumi-1 (Fig. 4C–E). Furthermore, western blot was performed to assess proteins associated with cell cycle and apoptosis. Knockdown of *SMIM3* resulted in an increase in cleaved-PARP and p27, and a significant decrease in Cyclin D1 and CDK4 (Fig. 4F–H).

### Identification of DEGs related to SMIM3 and enrichment analysis of DEGs

**Fig. 5:**
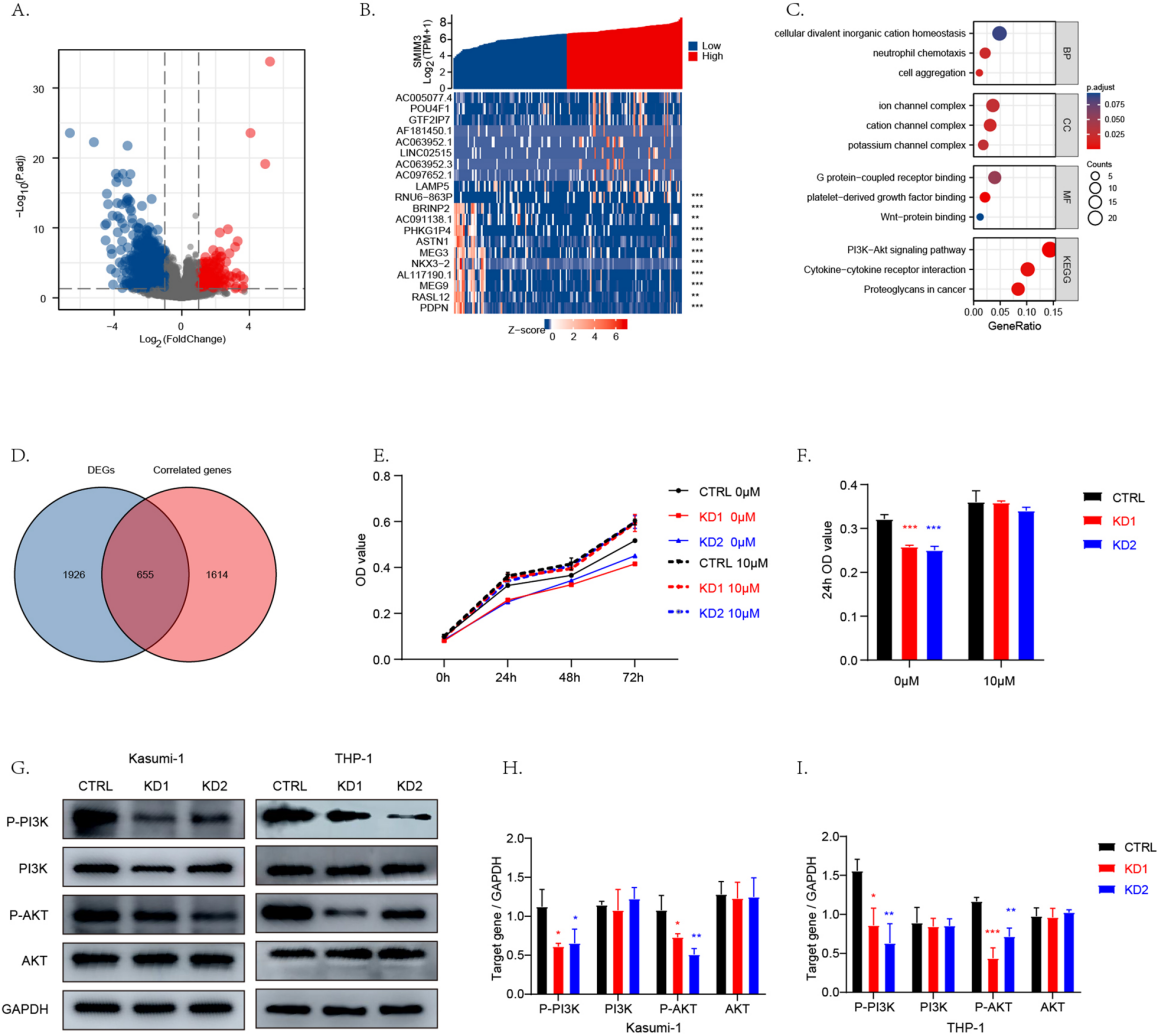
GO/KEGG enrichment analysis of subjects with high or low *SMIM3* expression in the TCGA-LAML dataset. **A** Volcano map of the DEGs, including 231up-regulated genes and 1889 down-regulated genes. **B** Heat map showing the top ten up-regulated and the top ten down-regulated genes. The samples are shown on the X-axis, and the DEGs are shown on the Y-axis. **D** Venn diagram of the overlap among the DEGs and correlated genes. **C** GO and KEGG enrichment analysis of the co-expressed genes of DEGs and correlated genes. MF, molecular function. CC, cellular component. BP, biological process. Different categories were shown on the Y-axis, and the X-axis reflected the percentage of DEGs. PI3K-AKT signaling pathway is critical for *SMIM3*-mediated changes. **G** Western blot analysis of PI3K-AKT signaling pathway in *SMIM3*-KD cells and controls. **H**, **I** Densitometric analysis of the WB signals. **E** SC79 (10 mM) reversed the inhibitory effect of *SMIM3* knockdown on the proliferation of Kasumi-1, **F** at 24 h. CTRL, control; KD, knockdown; ***P < 0.001 compared with CTRL cells; Error bars indicate the standard deviation

As shown in volcano map (Fig. 5A), 231 genes (red dots) were significantly upregulated, and 1889 genes (blue dots) were downregulated (|log2(FC)|> 1 & p.adj < 0.05). The top ten up-regulated DEGs and top ten down-regulated DEGs between the high- and low- *SMIM3* groups were displayed in the heat map (Fig. 5B). Moreover, we screened out some genes that had a high correlation with *SMIM3* (p < 0.5,|r|> 0.3). The Venn diagram (Fig. 5D) showed the overlap among the DEGs and correlated genes. Further GO and KEGG pathway enrichment analyses of these 655 co-expressed genes were carried out (Fig. 5C). Cellular components (CC) associated with high *SMIM3* included cation channel complex, ion channel complex, potassium channel complex. Molecular function (MF) associated with high *SMIM3* included Wnt-protein binding, platelet-derived growth factor binding, G protein-coupled receptor binding. KEGG analysis revealed that DEGs were mainly involved in phosphoinositide 3-kinase (PI3K)−AKT signaling pathway, proteoglycans in cancer and cytokine-cytokine receptor interaction. For this study, we chose PI3K −AKT for in vitro and in vivo validation.

### PI3K- AKT signaling pathway was associated with the function of SMIM3 in AML cells

Based on the above findings, we screened signaling pathways that could be involved in the function of *SMIM3*. As shown in Fig. 5G, phosphorylation of PI3K and AKT were notably decreased in *SMIM3* knockdown cells. To further validate the effect of *SMIM3* on PI3K-AKT signaling pathway, the AKT agonist SC79 was used to treat the SMIM3-KD cells of Kasumi-1. The addition of the AKT agonist (SC79) significantly reversed the inhibitory effect of *SMIM3* knockdown on the proliferation of AML cells (Fig. 5E, F).

### Knockdown of SMIM3 inhibited the growth of tumor tissues in vivo

**Fig. 6:**
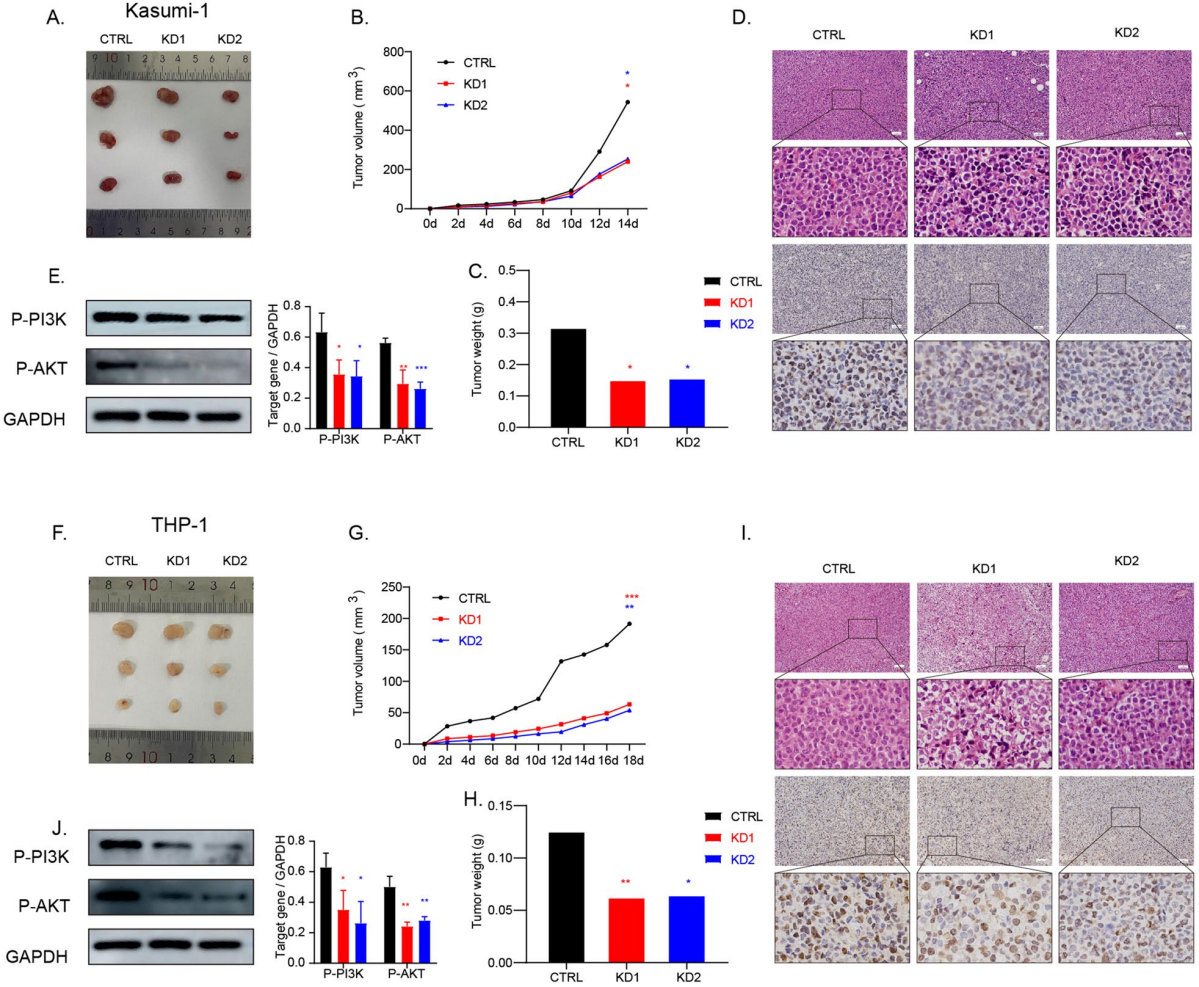
*SMIM3* knockdown reduced the proliferation of AML cells in nude mice by inhibiting PI3K-AKT signaling pathway. **A**–**D**, **F**–**I** *SMIM3*-KD inhibits tumor growth in Kasumi-1 and THP-1 cells. **E**, **J** The expression of PI3K-AKT signaling pathway proteins in tumor tissues by Western blot. CTRL, control; KD, knockdown; ***P < 0.001 compared with CTRL cells

To validate the effects of *SMIM3* from the in vitro experiments and the involvement of PI3K-AKT signaling pathways, we applied a vivo xenograft model. Our study showed that in Kasumi-1and THP-1 cells, the knockdown of *SMIM3* significantly suppressed tumor growth compared to that in the control group. The tumor formation in nude mice revealed that the volume and weight of tumor in the experimental group with *SMIM3* knockdown were significantly reduced than that of the control group (Fig. 6A–C, F–H). We also performed Ki67 immunohistochemical staining and *HE* staining to evaluate tumor cell proliferation (Fig. 6D, I). The result of Ki67 and HE staining confirmed that the knockdown of *SMIM3* inhibited tumor growth. Western blot showed that p-AKT and p-PI3K were significantly decreased (Fig. 6E, J). These changes in vivo were consistent with the results in vitro.

### Correlations between SMIM3 expression and sensitivity to therapy drugs

**Fig. 7:**
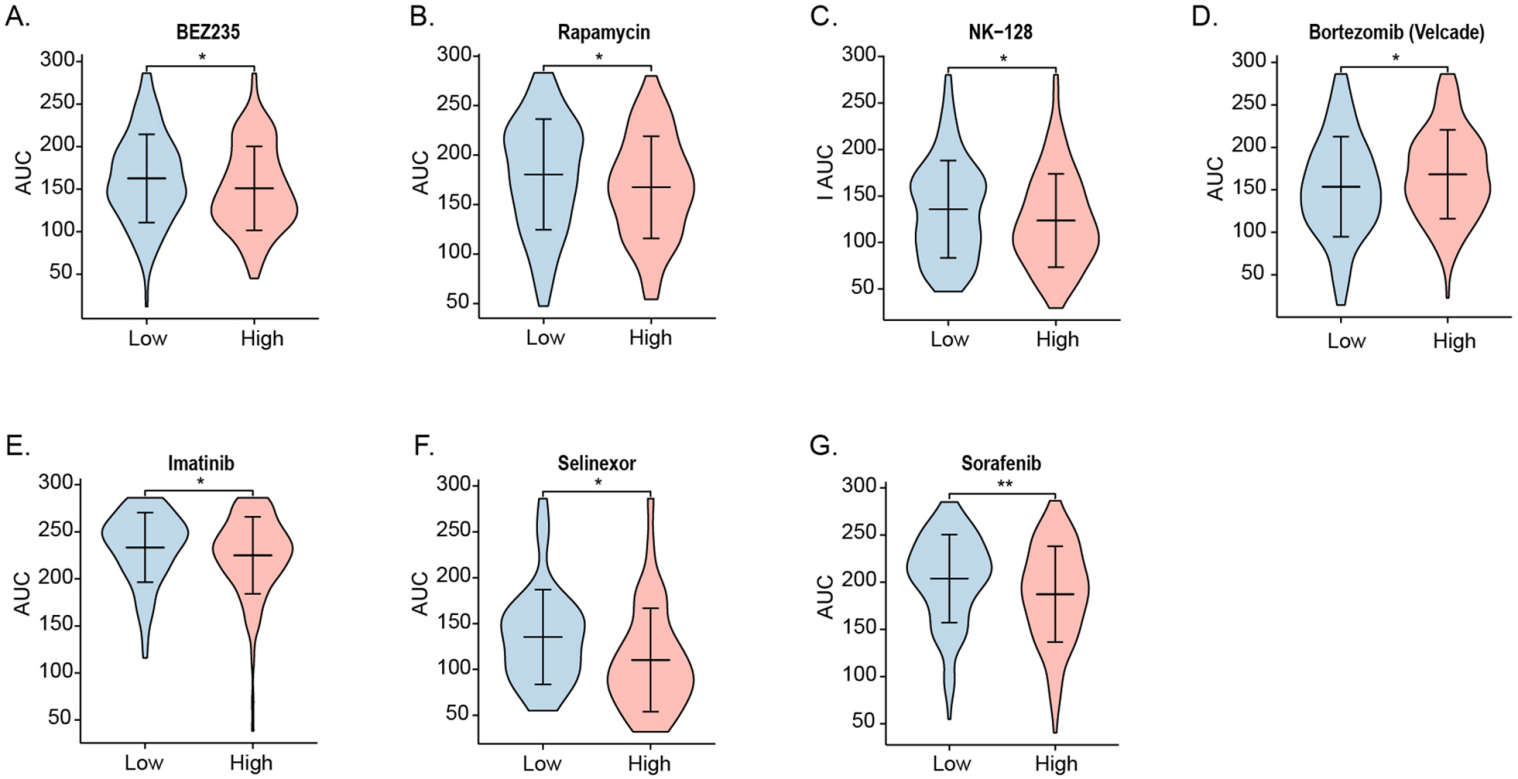
The relationship between drugs sensitivity and the expression level of *SMIM3*

Molecular-targeted therapy is a common treatment for AML patients. Moreover, the purpose of our study was to find genes associated with the prognosis of AML, in order to further guide the treatment. Thus, based on a previous study [38], we compared the distribution of drugs sensitivity represented by the area under the curve (AUC) with the expression level of *SMIM3*. Results indicated that patients with high expression of *SMIM3* were more sensitive to BEZ235, Imatinib, INK–128, Rapamycin, Selinexor, and Sorafenib (Fig. 7A–C, E–G). Patients with low expression of *SMIM3* were more sensitive to Bortezomib (Fig. 7D). BEZ235, INK-128 and Rapamycin belong to the family of PI3K-AKT-mTOR inhibitor. Imatinib belongs to the family of tyrosine kinase inhibitor. Selinexor is a small molecule Exportin-1 (XPO1) inhibitor. Sorafenib belongs to the family of RTK inhibitor. Bortezomib is a proteasome inhibitor.

## Discussion

This study investigated the prognostic value and molecular mechanism of *SMIM3* in adult AML. Based on the bioinformatics analyses and our clinical data, we found that adult AML patients showed a significant increase in the expression level of *SMIM3* compared with normal controls. In NK-AML, the high *SMIM3* expression was independently associated with a poor prognosis. To highlight the function of *SMIM3* in AML, we further explored the critical effects of *SMIM3* on cell behaviors through both in vivo and in vitro experiments. Our results suggested that the knockdown of *SMIM3* could inhibit cell proliferation and colony formation, arrest cell cycle progression and promote apoptosis. The effect on cell proliferation may occur through downregulating PI3K-AKT signaling pathway.

We performed a pan‐cancer expression analysis of *SMIM3* and showed that *SMIM3* was highly upregulated in 8 cancers and commonly downregulated in 16 cancers. This suggests that *SMIM3* has complex regulatory roles, and can act as either a potential oncogene or a tumor suppressor gene in different cancer types. The high expression of *SMIM3* in AML was also further validated in GEO database, ZZU cohort and cell lines.

Current studies found multiple connections between *SMIM3* and various diseases, including pheochromocytoma [27], 5q- syndrome of MDS [28] and radiation exposure [29]. There are few studies on the biological functions of *SMIM3* in different cancers. A study revealed that *SMIM3* may be associated with poor prognosis in oral squamous cell carcinoma [30], which needs to be further verified. Interestingly, membrane proteins with a similar structure to *SMIM3* (such as the minK family [39], the γ subunit of the Na, K-ATPase [40, 41] and phospholamban [42]) are thought to play a regulatory role in ion channel subunits, suggesting that *SMIM3* may have a similar role [28]. However, there has not been any in-depth study in the function of *SMIM3* yet. Until now, the upstream and downstream mechanisms of *SMIM3* expression remain unclear. Based on our current results, we would like to further investigate molecular mechanisms in future studies. What’s more, no SMIM family gene has been studied in AML. But several genes do play a role in other cancers. This suggests that SMIM family may play a role in cancers. Further studies should be carried out to define the function of these genes in AML.

The wide application of the existing risk stratification diagnosis and treatment has improved the prognosis of AML patients. Even though the risk stratification system was associated with adult AML prognosis in our cohort, no significant survival difference was found in NK-AML. This indicated the need to improve the risk stratification for NK-AML patients. Our study revealed that high expression of *SMIM3* was associated with worse OS in NK-AML. This result was confirmed in several databases. Moreover, multivariate survival analysis indicated that high *SMIM3* expression and transplant were independent prognostic factors for unfavorable OS in ZZU NK-AML cohort. NK-AML patients without transplant had a poor prognosis in H- *SMIM3* group, but there was no significant difference in the allo‐HSCT subgroup. This result suggested that allo‐HSCT may be an effective way to overcome the adverse impact of *SMIM3*. Collectively, these results suggested that *SMIM3* might be a novel prognostic marker for NK-AML patients.

The abnormally high expression and poor prognosis suggested the biological functions of *SMIM3* in AML. Our study provided further evidence that *SMIM3* affected the proliferation of AML cells through apoptosis and cell cycle regulation both in vitro and in vivo. The results of IF revealed that *SMIM3* was mainly localized in the nucleus. The knockdown of *SMIM3* caused G0/G1 cell cycle arrest via the p27/Cyclin D1-CDK4 pathway. The induction of p27, a cyclin dependent kinase inhibitor, caused cell cycle progression at the G0/G1 phase [43–46]. Also, G0/G1 phase is regulated by CDK4 and Cyclin D1 [47–49]. The upregulation of p27 could inhibit the activity of Cyclin D1-CDK4 [45]. To further understand the mechanism of the biological functions, we studied the changes in critical signaling pathways related to proliferation, apoptosis and metabolism based on KEGG analysis in AML cell lines. The PI3K-AKT signaling pathway plays a central role in metabolism. It regulates crucial functions including proliferation, differentiation, and survival [50]. The activation of this pathway was suggested to be associated with adverse prognosis [51]. Our results showed that the phosphorylated PI3K-AKT was reduced in the *SMIM3*-KD cells. These suggested that *SMIM3* could modulate the growth and survival of AML cells by regulating the PI3K-AKT signaling pathway. Based on a previous database, we found that the H-*SMIM3* group was more sensitive to PI3K-AKT-targeted drugs. These were consistent with KEGG, in vitro and in vivo results. Additionally, H-*SMIM3* group was also more sensitive to various first-line and novel drugs, including Imatinib, Selinexor, Sorafenib and Bortezomib. These results provided the basis for the application of targeted drugs, which could reduce the chance of relapses and drug resistance. In addition, GO and KEGG pathway enrichment analyses also found that cation channel complex, ion channel complex and potassium channel complex were associated with high *SMIM3.*

There are still some drawbacks in our study. Firstly, our study had the inherent limitations of any retrospective study. Secondly, our results need to be further verified in multicenter large sample prospective cohort studies.

## Conclusions

In summary, this was the first study to elucidate the significance of *SMIM3* in adult AML. Our study indicated that high expression level of *SMIM3* was associated with poor OS in adults NK-AML patients. In addition, knockdown of *SMIM3* inhibited proliferation and cell cycle progression, and induced apoptosis of AML cell lines through downregulating PI3K-AKT signaling pathway. These findings provided evidence that *SMIM3* may serve as a potential prognostic marker and personalized treatment target for AML in the future.

## Supplementary Information

**Additional file 1: Table S1.** Sequences of primers and probes used in this study. **Table S2.** Relationship between Transcription Level of SMIM3 and Clinical Characteristics in Normal Karyotype AML. **Table S3**. The catalog number of reagents.

**Additional file 2: Figure S1.** Overall survival (OS) of adult subjects with AML according to SMIM3. OS of 236 AML subjects in ZZU.(We didn't find our Figure S1 in the file, do we need to upload the Figure S1 again?) **Figure S2.** Immunohistochemical analysis of tissues in tumor tissues.

**Additional file 3.**

## Data Availability

The datasets generated and analyzed during the current study are not publicly available due to patient privacy considerations, but are available from the corresponding author on reasonable request (Shujuan Wang: fccwangsj1@zzu.edu.cn).

## Abbreviations

*SMIM3*: Small integral membrane protein 3;
HSC: Hematopoietic stem cell;
BMMC: Bone marrow mononuclear cells
GTEx: Genotype-Tissue Expression
TCGA: The cancer genome atlas
GEO: Gene expression omnibus
WBC: White blood cell count
HGB: Hemoglobin
PLT: Platelet
PB: Peripheral blasts
BM: Bone marrow
Ara-C: Standard-dose cytarabine
DNR: Daunorubicin
CR: Complete remission
OS: Overall survival
RT-qPCR: Real-time quantitative polymerase chain reaction
RPMI: Roswell park memorial institute
FBS: Fetal bovine serum
P/S: Penicillin/streptomycin
MOI: Multiplicity of infection
IF: Immune fluorescence
EDTA: Ethylene diamine tetraacetic acid
PBS: Phosphate-buffered saline
PFA: Paraformaldehyde
RT: Room temperature
PMSF: Phenylmethylsulfonylfluoride
PAGE: Polyacrylamide gel electrophoresis
PVDF: Polyvinylidene difluoride
CCK8: Cell counting kit-8
ROC: Receiver operating characteristic
*HR*: Hazard ratio
*CI*: Confidence interval
*IQR*: Inter quartile range
