# Supplementary material for: Downregulation of SMIM3 inhibits growth of leukemia via PI3K-AKT signaling pathway and correlates with prognosis of adult acute myeloid leukemia with normal karyotype
Source: J Transl Med. 2022 Dec 22;20:612. doi: 10.1186/s12967-022-03831-8 (PMC9783723; doi:10.1186/s12967-022-03831-8)
Supplement: Supplementary file 1 — Additional file 1: Table S1. Sequences of primers and probes used in this study. Table S2. Relationship between Transcription Level of SMIM3 and Clinical Characteristics in Normal Karyotype AML. Table S3. The catalog number of reagents. [file 12967_2022_3831_MOESM1_ESM.docx]

| **Table S1． Sequences of primers and probes used in this study** | |
| --- | --- |
| Name | Sequence (5'-3') |
| *SMIM3*-Forward primer | TCATCATGACCTCGTTGTTGCT |
| *SMIM3*-Reverse primer | CTCAAACAGCCCCACTAAGGAT |
| *SMIM3*-Probe | FAM-ACTGCAGTAATCATCTATCGCATGCGGAC-BHQ |
| *ABL1*-Forward primer | CCGCTGACCATCAATAAGGAA |
| *ABL1*-Reverse primer | GATGTAGTTGCTTGGGACCCA |
| *ABL1*-Probe | FAM-CCATTTTTGGTTTGGGCTTCACACCATT-TAMARA |

**Table S2. Relationship between Transcription Level of *SMIM3* and Clinical Characteristics in Normal Karyotype AML.**

| Characteristic | Total | L-SMIM3 | H-SMIM3 | *P*-value |
| --- | --- | --- | --- | --- |
|  | N=95 | N=74 | N=21 |  |
| Age, years | 44.5 ±15.0 | 45.1 ± 15.6 | 42.6 ± 13.0 | 0.469 |
| ≥55, n(%) | 21 (22.1%) | 18 (24.3%) | 3 (14.3%) | 0.389 |
| Female | 54 (56.8%) | 43 (58.1%) | 11 (52.4%) | 0.827 |
| **Risk, group n(%)** |  |  |  | **0.01** |
| Poor | 38 (40.0%) | 33 (44.6%) | 5 (23.8%) |  |
| Intermediate | 41 (43.2%) | 26 (35.1%) | 15 (71.4%) |  |
| Favorable | 16 (16.8%) | 15 (20.3%) | 1 (4.76%) |  |
| FAB, n (%) |  |  |  | 0.463 |
| M0 | 13 (13.7%) | 10 (13.5%) | 3 (14.3%) |  |
| M1 | 7 (7.37%) | 6 (8.11%) | 1 (4.76%) |  |
| M2 | 37 (38.9%) | 32 (43.2%) | 5 (23.8%) |  |
| M4 | 9 (9.47%) | 6 (8.11%) | 3 (14.3%) |  |
| M5 | 24 (25.3%) | 16 (21.6%) | 8 (38.1%) |  |
| M7 | 5 (5.26%) | 4 (5.41%) | 1 (4.76%) |  |
| WBC, ×109/L | 34.9 [8.80;94.1] | 29.7 [8.04;88.2] | 59.5 [18.2;98.4] | 0.262 |
| HGB, g/L | 82.2 ± 23.8 | 82.1 ± 25.7 | 82.6 ± 15.9 | 0.912 |
| PLT, ×109/L | 47.0 (21.5-80.5) | 47.5 (20.2-84.8) | 43.0 (27.0-64.0) | 0.771 |
| LDH U/L | 388 (257-828) | 394 (288-807) | 386 (230-919) | 0.754 |
| PB(%) | 74.0 (36.0-90.0) | 70.5 (28.0-89.8) | 79.0 (42.0-90.0) | 0.407 |
| BM-blast(%) | 72.0 (48.0-84.2) | 66.8 (42.8-84.3) | 80.5 (68.0-83.6) | 0.053 |
| Mutations, n(%) |  |  |  |  |
| *FLT3* | 27 (28.4%) | 17 (23.0%) | 10 (47.6%) | 0.053 |
| ***FLT3_ITD*** | **26 (27.4%)** | **16 (21.6%)** | **10 (47.6%)** | **0.037** |
| *FLT3_TKD* | 2 (2.11%) | 2 (2.70%) | 0 (0.00%) | 1 |
| *RUNX1* | 3 (3.16%) | 2 (2.70%) | 1 (4.76%) | 0.532 |
| *WT1* | 65 (68.4%) | 52 (70.3%) | 13 (61.9%) | 0.644 |
| *MLL* | 1 (1.10%) | 0 (0.00%) | 1 (5.00%) | 0.22 |
| *TET2* | 52 (54.7%) | 41 (55.4%) | 11 (52.4%) | 1 |
| *CEBPA* | 27 (28.4%) | 23 (31.1%) | 4 (19.0%) | 0.421 |
| *NPM1* | 24 (25.3%) | 15 (20.3%) | 9 (42.9%) | 0.069 |
| *ASXL1* | 22 (23.2%) | 20 (27.0%) | 2 (9.52%) | 0.142 |
| *NRAS* | 14 (14.7%) | 11 (14.9%) | 3 (14.3%) | 1 |
| *KIT* | 1 (1.05%) | 1 (1.35%) | 0 (0.00%) | 1 |
| *JAK2* | 1 (1.05%) | 1 (1.35%) | 0 (0.00%) | 1 |
| *DNMT3A* | 19 (20.0%) | 12 (16.2%) | 7 (33.3%) | 0.12 |
| *U2AF1* | 11 (11.6%) | 9 (12.2%) | 2 (9.52%) | 1 |
| *IDH2* | 8 (8.42%) | 8 (10.8%) | 0 (0.00%) | 0.193 |
| *IDH1* | 10 (10.5%) | 7 (9.46%) | 3 (14.3%) | 0.687 |
| *SRSF2* | 5 (5.26%) | 4 (5.41%) | 1 (4.76%) | 1 |
| *ETV6* | 2 (2.11%) | 2 (2.70%) | 0 (0.00%) | 1 |
| *TP53* | 2 (2.11%) | 1 (1.35%) | 1 (4.76%) | 0.395 |
| *EZH2* | 1 (1.05%) | 1 (1.35%) | 0 (0.00%) | 1 |
| *SETBP1* | 2 (2.11%) | 1 (1.35%) | 1 (4.76%) | 0.395 |
| *CBL* | 3 (3.16%) | 2 (2.70%) | 1 (4.76%) | 0.532 |
| *PHF6* | 2 (2.11%) | 1 (1.35%) | 1 (4.76%) | 0.395 |
| *ETO* | 0 (0.00%) | 0 (0.00%) | 0 (0.00%) |  |
| *CBFβ* | 0 (0.00%) | 0 (0.00%) | 0 (0.00%) |  |
| *ZRSR2* | 0 (0.00%) | 0 (0.00%) | 0 (0.00%) |  |
| *SF3B1* | 0 (0.00%) | 0 (0.00%) | 0 (0.00%) |  |
| CR | 63 (66.3%) | 50 (67.6%) | 13 (61.9%) | 0.824 |
| Transplant | 0.19 (0.39) | 0.18 (0.38) | 0.24 (0.44) | 0.557 |

WBC, white blood cell counts; HGB, hemoglobin; PLT, platelet; LDH, lactate dehydrogenase; BM, bone marrow; PB, peripheral blood; CR, complete remission;

**Table S3 The catalog number of reagents**

| Reagent | Company | Catalog number |
| --- | --- | --- |
| Fetal bovine serum | Gibco, Billings, MT, USA | 10099141C |
| SC79 | Beyotime, Shanghai, China | SF2730 |
| TRIzol Reagent | Invitrogen, Carlsbad, CA, USA | 15596026 |
| High Capacity cDNA Reverse Transcription Kit | Applied Biosystems, Foster City, CA, USA | 4368813 |
| Triton-X-100 | Beyotime, Shanghai, China | P0096 |
| 5% BSA | Solaibao Biotechnology, Beijing, China | SW3015 |
| DAPI | Solaibao Biotechnology, Beijing, China | C0065 |
| RIPA lysis buffer | Beyotime, Shanghai, China | P0013C |
| Protein phosphatase inhibitor | Biomed, Beijing, China | PA113-01 |
| phenylmethylsulfonylfluoride | Biomed, Beijing, China | PA116-01 |
| Super ECL Prime | US EVERBRIGHT, Suzhou, China | S6008 |
| Cell Counting Kit-8 | Dojin Laboratories, Kumamoto, Japan | CK04 |
| MethoCult™ H4230 | STEMCELLTM TECHNOLOGIES, Vancouver, British Columbia, Canada | 4230 |
| Cell Cycle Staining Kit | Lianke Biotechnology, Hangzhou, China | CCS012 |
| Annexin V-APC/PI Apoptosis Kit | US EVERBRIGHT, Suzhou, China | A6030 |
| GAPDH | Cell Signaling Technology | 5174 |
| goat anti-rabbit IgG horseradish peroxidase (HRP) | Zhongshan Golden Bridge Biotechnology, Beijing, China | ZB-2301 |
| antibody Cy3 conjugated Goat Anti-Rabbit IgG (H+L) | Servicebio, Wuhan, China | GB21303 |
| goat anti-rabbit IgG-HRP | Servicebio, Wuhan, China | G1213 |
| anti-SMIM3 Polyclonal Antibody | Thermo Fisher Scientific, Waltham, MA, USA | PA5-67381 |
| cleaved-PARP | Cell Signaling Technology | 5625 |
| cleaved caspase 3 | Cell Signaling Technology | 9664 |
| p27 Kip1 | Cell Signaling Technology | 3686 |
| Cyclin D1 | Cell Signaling Technology | 2978 |
| CDK4 | Cell Signaling Technology | 12790 |
| p-AKT | Cell Signaling Technology | 4060 |
| AKT | Cell Signaling Technology | 4691 |
| PI3K | Cell Signaling Technology | 4257 |
| p-PI3K | Affinity Biosciences LTD, Jiangsu, China | AF3241 |
| Ki67 | Servicebio, Wuhan, China | GB111499 |
| cleaved caspase 3 | Cell Signaling Technology | 9664 |
| Anti -Cleaved- Caspase-3 Rabbit pAb | Servicebio, Wuhan, China | GB11532 |
