# Supplementary material for: Downregulation of SMIM3 inhibits growth of leukemia via PI3K-AKT signaling pathway and correlates with prognosis of adult acute myeloid leukemia with normal karyotype
Source: J Transl Med. 2022 Dec 22;20:612. doi: 10.1186/s12967-022-03831-8 (PMC9783723; doi:10.1186/s12967-022-03831-8)

**Figure S1.**

Overall survival (OS) of adult subjects with AML according to *SMIM3*. OS of 236 AML subjects in ZZU


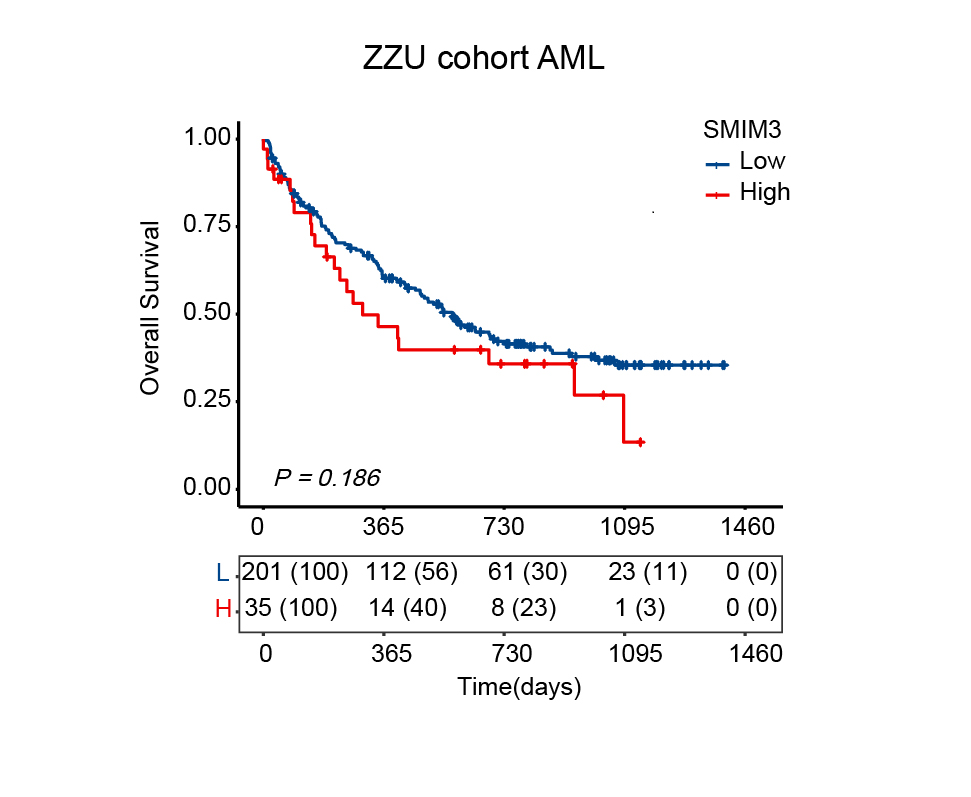


**Figure S2.**

Immunohistochemical analysis of tissues in tumor tissues.


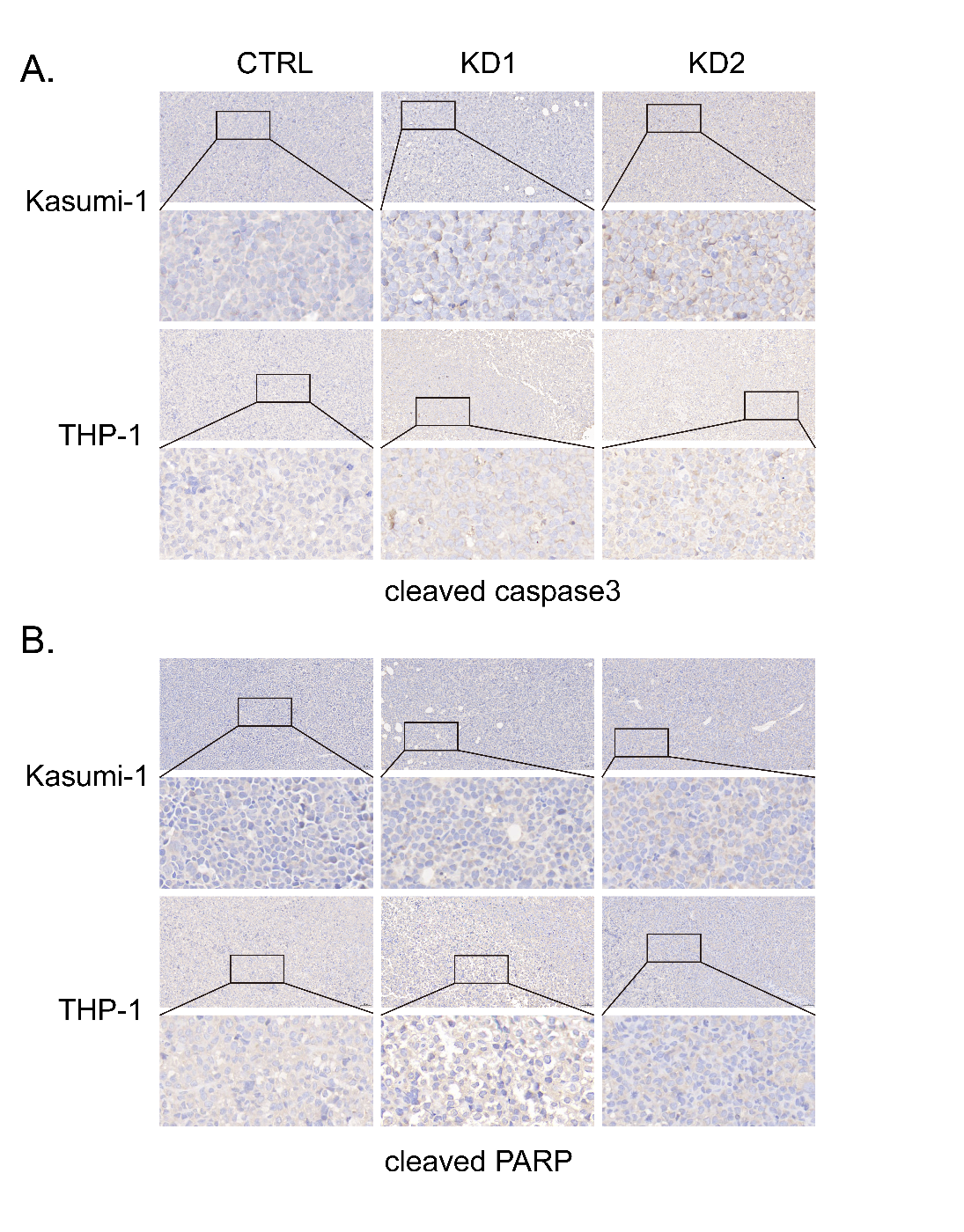

Supplement: Supplementary file 2 — Additional file 2: Figure S1. Overall survival (OS) of adult subjects with AML according to SMIM3. OS of 236 AML subjects in ZZU.(We didn't find our Figure S1 in the file, do we need to upload the Figure S1 again?) Figure S2. Immunohistochemical analysis of tissues in tumor tissues. [file 12967_2022_3831_MOESM2_ESM.docx]
